# Influence of Melatonin on the Proliferative and Apoptotic Responses of the Prostate under Normal and Hyperglycemic Conditions

**DOI:** 10.1155/2015/538529

**Published:** 2015-07-30

**Authors:** Marina G. Gobbo, Nishtman Dizeyi, Per-Anders Abrahamsson, Per-Anders Bertilsson, Viviane Sanches Masitéli, Eloisa Zanin Pytlowanciv, Sebastião R. Taboga, Rejane M. Góes

**Affiliations:** ^1^Department of Cell Biology, Institute of Biology, UNICAMP, Avenue Bertrand Russel, 6109 Campinas, SP, Brazil; ^2^Department of Clinical Sciences, Division of Urological Research, Skåne University Hospital, Lund University, 205 02 Malmö, Sweden; ^3^Department of Biology, Institute of Biosciences, Humanities and Exact Sciences, UNESP, São José do Rio Preto, SP, Brazil

## Abstract

The antitumor properties of melatonin (MLT) are known for prostate cancer cells. This study investigated whether MLT affects prostate maturation and interferes with tissue injuries induced by diabetes. MLT was administered to Wistar rats from 5 weeks of age in the drinking water (10 *μ*g/kg b.w.), and diabetes was induced at the 13th week by streptozotocin (4.5 mg/100g b.w., i.p.). The animals were euthanized in the 14th and 21st weeks. MLT reduced the immunostained cells for androgen receptor (AR) by 10% in younger rats. Diabetes decreased cell proliferation and increased apoptosis. MLT treatment impeded apoptosis (*p* = 0.02) and augmented proliferation (*p* = 0.0008) and PCNA content in prostate following long-term diabetes due to restoration of testosterone levels and expression of melatonin receptor type 1B. The effect of MLT (500 *µ*M, 5 mM, and 10 mM) on androgen-dependent (22Rv1) and androgen-independent (PC3) cancer cells and human prostate epithelial cells (PNTA1) under normal and hyperglycemic conditions (HG, 450 mg/dL) was analyzed. Contrary to PNTA1 and 22Rv1 cells, MLT improved the proliferation of PC3 cells in hyperglycemic medium. The combined data indicated that MLT had proliferative and antiapoptotic effects in prostate cells subjected to HG levels and it seems to involve specific MLT pathways rather than AR.

## 1. Introduction

Melatonin (MLT) is an indoleamine produced by the pineal gland that controls the circadian cycle and acts as a neuromodulator, cytokine, and biological response modifier [1]. This hormone is widely known as an adjustor of the reproductive physiology to the environmental light in seasonally dependent mammals [2]. MLT also has antioxidant [3–6] and anti-inflammatory properties [7, 8]. The cellular action of MLT is mediated by three receptor subtypes (MT1, MT2, and MT3) coupled to G proteins. MLT signaling may also be modulated by the activation of a series of nuclear receptors referred to as retinoid Z receptors *α* and *β* [9, 10]. Depending on the concentration, MLT can act directly in the cytosol through calmodulin and quinone reductase 2 [11–13].

Data from Gilad et al. [14] indicated the presence of MLT receptors in the rat prostate. The inhibition of xenografted prostate tumor growth by MLT treatment has been reported in rodents, and the antiproliferative action of MLT occurred via activation of the MT1 receptor with consequent attenuation of calcium influx induced by sex steroids [15–17]. The antitumor action of MLT in prostate cancer cell lines has been attributed to changes in cell cycle, androgen receptor (AR) translocation, and inhibition of angiogenesis through reduced expression of factors that act under hypoxic conditions, such as hypoxia-inducible factor 1*α* (HIF-1*α*) [18–25]. MLT also exerts a proapoptotic effect in tumor cells via p38 and c-jun terminal kinases (JNK); however, this effect is independent of extracellular signal-regulated kinase (ERK) activation [26]. This hormone has been associated with increased sensitivity to some chemotherapeutic drugs [27]. Moreover, androgen-independent cell lines, such as PC3 and DU145, are less sensitive to MLT compared to the androgen-dependent cells, such as LNCaP, CW22Rv1, and RWPE-1 [28–30].

Because MLT has an antigonadotrophic effect in humans and rodents by inhibiting testosterone synthesis in the testis [31, 32], it is difficult to discriminate the direct influence of this hormone in androgen-dependent organs, such as the prostate. Few studies have considered the effects of MLT on prostate* in vivo*, especially during sexual maturation.

The effects of diabetes on prostate histophysiology have been investigated in rodents [33–42]. The alterations include atrophy, impaired secretory activity, reduced cell proliferation, an increased number of apoptotic and epithelial basal cells, extracellular matrix remodeling, impaired androgen sensitivity, and phenotypic changes in stromal cells [34, 36, 38, 40, 42–48]. The prostatic response to diabetes has been related to the reduction of serum testosterone levels and lack of insulin, typical of this metabolic disorder [48, 49].

The diabetic condition is associated with a large production of reactive oxygen species resulting from hyperglycemia. The effectiveness of therapy with different antioxidants against oxidative stress caused by diabetes has been extensively investigated experimentally [50–55]. Treatment with ascorbic acid normalized the increased glutathione S-transferase (GST) activity and reduced epithelial apoptosis in the prostate after one month of experimental diabetes induced by streptozotocin [53]. MLT is a potent antioxidant, and its synthesis is impaired under hyperglycemia [56]; thus, it is worthwhile to investigate the efficacy of MLT treatment under diabetic conditions and its influence on prostate cells under hyperglycemia.

This study examined whether pre- and cotreatment with MLT interferes with tissue damage induced in the prostate of Wistar rats by experimental diabetes, particularly in terms of proliferative activity, apoptosis, and AR expression. In this study we report for the first time the effects of continuous use of this neurohormone during sexual maturation on these processes and prostate histology. We also compared the influence of MLT under hyperglycemic condition on the proliferation and apoptosis of androgen-dependent (CW22Rv1) and androgen-independent (PC3) cancer cells and human prostate epithelial cells (PNTA1).

## 2. Materials and Methods

### 2.1. *In Vivo *Experimental Design

Male Wistar rats (*n* = 80) were obtained from the breeding house of São Paulo State University (Botucatu, SP, Brazil) in the 5th week of life (weaning). This experiment was conducted according to the ethical principles adopted by the Guide for the Care and Use of Laboratory Animals published by the US National Institutes, and all procedures were approved by the Ethics Committee on Animal Use of IBILCE/UNESP (Protocol 051/2011 CEUA). The rats were kept in polyethylene cages with wood shaving substrate, subjected to light cycles (14 h of light and 10 h of darkness) and a temperature of approximately 25°C. Food (Presence,* In vivo*, Paulinia, SP, Brazil) and filtered water were provided* ad libitum*.

After one week of adaptation, the animals were weighed and randomly distributed into two experiments, with eight groups total (10 animals per group). The short-term experiment consisted of a control group (C1), a control treated with melatonin (M1), a one-week diabetic group (D1), and a one-week diabetic group treated with melatonin (MD1). The long-term experiment consisted of a control group (C2), a control treated with melatonin (M2), an eight-week diabetic group (D2), and an eight-week diabetic group treated with melatonin (MD2).

MLT administration (Sigma Chemical Co., St. Louis, MO, USA) was based on the procedures established by Wolden-Hanson et al. [57]. MLT was dissolved in ethanol and stored in aliquots at −80°C. Such conditions were standardized by application of various consumption preference and aversion tests, and MLT did not affect the amount of water consumed by rats. MLT was available to animals (groups M1, M2, MD1, and MD2) in drinking water (10 *μ*g/kg body weight in ethanol 0.001%/day) from 5 to 14 weeks old. The MLT intake per day of this investigation was based on an average daily water consumption of 80 mL/day/animal and an average body weight of 350 g. Water bottles were protected from light because MLT is a photosensitive molecule, and the liquid content was changed every day.

Diabetes was induced on the 8th week of MLT treatment (13 weeks old) in groups D1, D2, MD1, and MD2 by the intraperitoneal injection of 4.5 mg/100 g of body weight of streptozotocin (Sigma Chemical Co., Louis, MO, USA), diluted in 1 mL of 0.01 citrate buffer. This solution was injected after 24 h of fasting and anesthesia with ketamine and xylazine (0.1 mL/100 g of body weight). Control animals were injected with citrate buffer only.

Glucose levels were assessed two days after streptozotocin injection through the glucose monitor Accu-Chek (Roche Diagnostics, Mannheim, Germany) in the fingertips of the paws. Animals with glucose levels above 200 mg/dL were used in this study. Due to the higher water intake of diabetic animals, the MLT dose was corrected for groups D1, D2, MD1, and MD2 after the diagnosis of diabetes. Groups C1, M1, D1, and MD1 were euthanized at 14 weeks old, whereas groups C2, M2, D2, and MD2 were euthanized at 21 weeks old. Thus, there was a short administration of MLT for groups M1 and MD1 (9 weeks) and a prolonged treatment for groups M2 and MD2 (16 weeks). The rats were euthanized using CO_2_ inhalation and then decapitated for blood collection.

#### 2.1.1. Hormone Dosages

The blood samples were collected after decapitation and plasma was separated through centrifugation at 1,200 g and frozen at −80°C for analysis of testosterone levels. The measurements were performed by a capture/sandwich ELISA (antibody-antigen-antibody) using specific commercial kits (R&D Systems, Inc., Minneapolis, MN, USA), with a sensitivity of 14.0 pg/mL and a variation for interassays of 11.3 pg/mL. The readings were performed using an Epoch Multi-Volume Spectrophotometer System (BioTek Instruments, VT, USA).

#### 2.1.2. Light Microscopy

Ventral prostates were removed and weighed. Fragments of ventral prostate were fixed by immersion in 4% formaldehyde freshly prepared in phosphate buffer pH 7.2 and methacarn (1 : 3 : 6 of acetic acid, chloroform, and methanol) and embedded in Paraplast. The histological sections stained with hematoxylin-eosin were used for general morphological studies and immunocytochemical analysis. The sections were observed under a bright-field microscope (Olympus CH30) coupled with a charge-coupled camera. The digitization of selected microscopic fields and quantitative analyses were performed using an image analysis system (Image-Pro Plus Media Cybernetics, version 6.0 for Windows software, Bethesda, MD, USA).

#### 2.1.3. Immunohistochemistry

Immunocytochemical staining for proliferating cell nuclear antigen (PCNA), androgen receptor (AR), and melatonin receptor type 1B (MTR1B) was assessed using specific antibodies purchased from Santa Cruz Biotechnology (AR and PCNA, Santa Cruz, CA, USA) and Novus Biologicals (MTR1B, Novus Biologicals, Littleton, CO, USA). Sections were subjected to antigen retrieval in citrate buffer (10 mM, pH 6) for 20–40 min, followed by the blocking of endogenous peroxidase with 3% H_2_O_2_ in methanol or water (for MTR1B), and immersed at 3% normal bovine serum (for PCNA and MTR1B) or 5% dry milk (for AR) in PBS for 1 h to block nonspecific protein. After PBS washing, incubation with primary antibodies diluted in 1% BSA was carried out: AR (sc816, rabbit polyclonal, 1 : 100, overnight at 4°C), PCNA (sc56, mouse monoclonal, 1 : 100, 1 h at 37°C), and MTR1B (NLS932, rabbit polyclonal, 1 : 75, overnight at 4°C). Then, the tissue sections used for PCNA and AR were incubated at RT with a Polymer/peroxidase kit (Novolink Polymer, Novocastra, Norwell, MA, USA) for 1 h. The tissue sections used for MTR1B were incubated at 37°C with secondary antibody and then with avidin/biotin (ABC Staining Systems, Santa Cruz Biotechnology, CA, USA) for 45 min.

The reaction was detected with 0.1% diaminobenzidine (DAB) and 0.02% H_2_O_2_ in PBS, and the sections were counterstained with hematoxylin. Five animals per group and 3 prostatic fragments per animal were used to quantify the proliferation levels, AR-positive cells, and MTR1B positive areas. The sections were digitalized, and 30 contiguous fields were observed in the 40X objective. AR and PCNA-positive cells were quantified by counting the number of positive nuclei and dividing it by the total number of nuclei per visual field. Areas showing specific staining for MTR1B were evaluated using a 130-point reticulum and the marked area was counted as previously done [45]. Data were expressed as relative frequency (%).

#### 2.1.4. Detection of Apoptotic Cells

Apoptotic cells were detected* in situ* using the DNA fragmentation assay associated with cell death based on a Terminal deoxynucleotidyl transferase dUTP nick end labeling (TUNEL) reaction (TdT-Fragel-Calbiochem, CN Biosciences, La Jolla, CA, USA) following the manufacturer's instructions. The negative controls were obtained by omitting the incubation with TdT enzyme, and the slides were stained with hematoxylin. The quantification of apoptotic cells was performed in the same manner as the immunohistochemistry reaction for PCNA.

#### 2.1.5. Western Blotting Analysis

The PCNA protein content in the prostate samples was quantified by Western blotting. Prostate samples were homogenized at 4°C in cell lysis buffer (20 mM Tris-HCl, 150 mM NaCl, 1% Triton X100, 2% SDS) containing 100 mM phenylmethylsulfonyl fluoride, 100 mM sodium orthovanadate, and a protease inhibitor cocktail (1 : 1,000, Sigma, St. Louis, MO, USA Chemical Co.). Lysates were centrifuged at 13,000 g at 4°C for 15 min, the supernatants were collected, and the protein concentration was determined using the Bradford method [58]. Laemmli sample buffer with 5%  *β*-mercaptoethanol was added to equal amounts of protein (150 *μ*g). The samples were then separated by SDS-PAGE on 12% polyacrylamide Tris-glycine gels and electroblotted to nitrocellulose membrane (GE Healthcare) using Bio-Rad assay equipment (Bio-Rad, Hercules, CA, USA). Nonspecific proteins were blocked with 5% nonfat dry milk in TBST (10 mM Tris-HCl, pH 7.4, 150 mM NaCl, 0.2% Tween-20) for 1 h at room temperature, and the membranes were probed with primary antibody, PCNA (1 : 300) overnight at 4°C in 1% BSA in TBST. Then, the membranes were washed in TBST and incubated for 1 h at 4°C with the specific secondary horseradish peroxidase-conjugate antibody (1 : 200 in 1% BSA in TBST), followed by 3 washes in TBST. Antibody immunolabeling was revealed by an ECL chemiluminescent detection kit (Healthcare). *β*-actin was used as a control for protein expression. Densitometric analysis was performed using the Image J 1.34 software (Wayne Rasband, Research Services Branch, National Institute of Health, Bethesda, MD, USA).

### 2.2. *In Vitro *Experimental Design

Three human cell lines were used in this study: a nontumoral cell line (PNTA1) and two prostate cancer cell lines (CW22Rv1, herein referred to as 22Rv1 and PC3). 22Rv1 (CRL-2505, from xenograft line) and PC3 (CRL-1435, from bone metastasis) were purchased from the American Type Culture Collection (ATCC, Manassas, VA, USA). PNTA1 cells (#95012614) were obtained from the Health Protection Agency (UK). The cells were grown in RPM1640 medium containing 10% fetal bovine serum (FBS) and 1% penicillin-streptomycin (Life Technologies, Paisley, UK) in a humidified atmosphere of 95% air and 5% CO_2_ at 37°C. Cells were fed every 2-3 days and subcultured once they reached 70–80% confluence. The first phase of the investigation consisted of dose-related experiments with different melatonin concentrations (Sigma Chemical Co., St. Louis, MO, USA). Melatonin (MLT) was always freshly prepared in a 40% DMSO stock solution (Sigma Chemical Co., St. Louis, MO, USA) and diluted to different concentrations in the culture medium. After determining the correct range of MLT doses, the cell lines were preincubated in hyperglycemic medium with 450 mg/dL of glucose (DMEM; Invitrogen, Carlsbad, CA, USA) and then treated with MLT. Two different culture media were used because the 22Rv1 cells seeded in DMEM failed to attach properly. Thus, for the experiments with high glucose (HG) conditions, cells were cultured first with RPMI and then with DMEM with high glucose levels. The initial analysis of MLT effects using the light microscope revealed that 22Rv1 cells were more sensitive to indoleamine than the other cell types because more cells became detached when they were treated with 10 mM of MLT. Thus, a reduced MLT concentration (5 mM) was used for 22Rv1 cells, whereas PNTA1 and PC3 cells received 10 mM MLT. With this correction, a proportional cell density was maintained, which is crucial for the assays in this study. Thus, the following conditions were evaluated: in normal conditions (NC), the cells were plated with RPM1 medium and incubated with DMSO (at the same concentration of the highest dose of melatonin used). Melatonin treatment was performed in the following concentrations: 500 *μ*M, 5 mM, and 10 mM. Exposure to HG conditions was performed by replacing the RPM1 medium with hyperglycosylated DMEM (450 mg/dL) and incubating with the vehicle (DMSO). The concomitant exposure to HG and MLT was performed in the same manner as described separately above.

#### 2.2.1. Proliferation Assay

For this assay, 96-well flat-bottomed plates were used at a density of 3,000 cells/well with 100 *μ*L of medium. The cells were preincubated with HG DMEM for 1 day for the short-term experiment and 7 days for the long-term experiment. Then, the cells were treated with DMSO and MLT (500 *μ*M, 5 mM, or 10 mM) for an additional 1 and 2 days. The 3-(4,5-dimethylthiazol-2yl)-2,5-diphenyl tetrazolium bromide (MTT) is reduced by metabolically active cells, which results in the formation of purple formazan. A solution of 50% MTT in PBS was applied to each well, and the plate was incubated for 2 h in a humidified incubator at 37°C in an atmosphere of 5% CO_2_ protected from light. 100 *μ*L of isopropanol with 0.04 N HCl was added to each well and mixed by tapping gently on the plate. After 15 min, the absorbance was measured on an ELISA plate reader with a test wavelength of 570 nm and a reference wavelength of 630 nm. The results were expressed in absorbance values as a mean ± S.D. of the two experiments.

#### 2.2.2. Flow Cytometry

Cell cycle distribution (G0/G1, S, and G2/M) and apoptosis were analyzed by flow cytometry based on DNA content. 22Rv1, PNTA1, and PC3 cells were seeded in 6-well plates with a density of 100,000 cells and 1,000 *μ*L of RPM1. Some of the wells were preincubated with hyperglycemic DMEM for 24 h and then treated with MLT for an additional 24 h. The cells were washed in phosphate-buffered saline (PBS) without Ca^2+^ or Mg^2+^, followed by centrifugation (400 g, 5 min, 4°C). The fixation was performed in ice cold 70% ethanol followed by vortexing. The samples were stored at 4°C for 2 h and centrifuged again (400 g, 5 min, 4°C). For staining with propidium iodide (PI), cells were washed with cold PBS, centrifuged (300 g, 5 min, 4°C), and then suspended in a 500 *μ*g/mL PI solution (#11348639001; Roche, Mannheim, Germany) protected from the light at room temperature for 40 min. The samples were transferred to glass tubes (5 mL Falcon tubes) and kept on ice. The cells were gated, and the DNA content of at least 4,000 labeled cells was quantified with a FACSCalibur instrument (BD Bioscience, San Jose, CA, USA). During data analysis, doublet discrimination was performed using a two-parameter dot plot of FL2-Area versus FL2-Width. Data presented were analyzed using FlowJo 10.0.7 software (Treestar, Inc., Ashland, OR, USA). The percentage of fragmented DNA was considered an indirect measure of apoptosis.

#### 2.2.3. Statistical Analyses

Data were tested considering the assumptions of normality and homogeneity of variances according to the Shapiro-Wilk and Levene tests, respectively. The groups that have made such assumptions (parametric data) were compared by applying a* t*-test or one-way analysis of variance (ANOVA) followed by Tukey's test (*post hoc*). Data that did not fit these assumptions (nonparametric data) were compared by applying the Mann-Whitney* U* or Kruskal-Wallis test followed by Dunn's test (*post hoc*). Statistical analyses were performed between groups of the same experimental period and between different periods for the same treatment (for biometric data only) using the Statistica 9.0 software (Statsoft Inc., Tulsa, OK, USA). Data were expressed as a mean ± standard deviation, and *p* < 0.05 was considered statistically significant.

## 3. Results

### 3.1. *In Vivo *Experiment

#### 3.1.1. Physiological and Biometric Parameters

**Figure 1:**
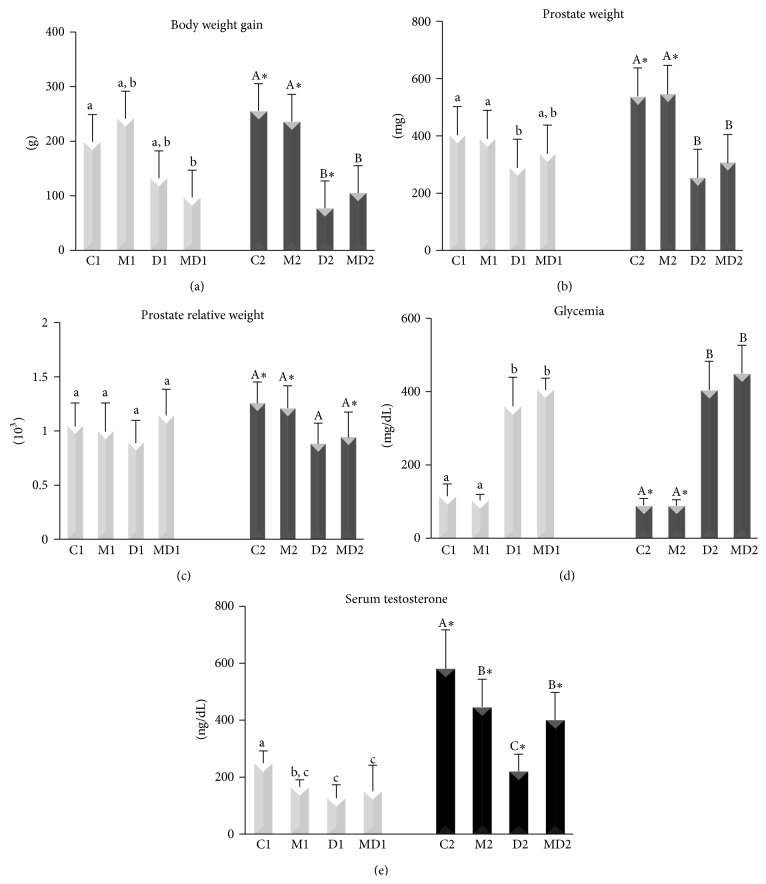
Mean and standard deviation of body weight gain (a), prostate wet weight (b), prostate relative weight (c), blood glucose levels (d), and serum testosterone (e). C1: short-term control; M1: short-term control treated with MLT; D1: short-term untreated diabetic; MD1: short-term diabetic treated with MLT; C2: long-term control; M2: long-term control treated with MLT; D2: long-term untreated diabetic; and MD2: long-term diabetic treated with MLT. Light bars: short-term experiment and dark bars: long-term experiment (*N* = 10 animals/group). Different lowercase letters indicate significant differences between experimental groups C1, M1, D1, and MD1 (parametric data: prostate weight, relative prostate weight, and serum testosterone; nonparametric data: body weight gain, glycemia), and different capital letters indicate significant differences between groups C2, M2, D2, and MD2 (parametric data: body weight gain, prostate weight, relative prostate weight, and serum testosterone; nonparametric data: glycemia). ^*∗*^Significant difference between experimental periods (*t*-test or Mann-Whitney* U *test).

Body weight gain (Figure 1(a)) did not vary among short-term experimental groups, except for a diminution in group MD1. Both diabetic groups from the longer duration experiment had smaller body weight gain, independently of MLT treatment (*p* < 0.0001). Prostate weight was not affected by MLT treatment under healthy conditions as well as in diabetes conditions in short- and long-term experiments (Figure 1(b)). MLT administration prevented partially prostate atrophy caused by short-term diabetes (*p* = 0.0025, Figure 1(b)), whereas this hormone was not able to prevent atrophy of the gland in two-month-old diabetic animals (*p* < 0.01). Ninety-one percent of the animals exhibited blood glucose levels of 404 mg/dL, as shown in Figure 1(d). Glycemia decreased by 18% from C1 to C2 (*p* = 0.026), and MLT did not change this parameter (Figure 1(d)). However, MLT affected the testosterone synthesis of healthy animals (Figure 1(e)) in both short- (decreased by 24%) and long-term (decreased by 34%) experiments. The serum androgen levels were also drastically decreased by induced diabetes (*p* < 0.005). Group MD2 exhibited higher levels of this hormone compared to group D2 (Figure 1(e)).

#### 3.1.2. Expression of the Androgen Receptor Using Immunocytochemistry

**Figure 2:**
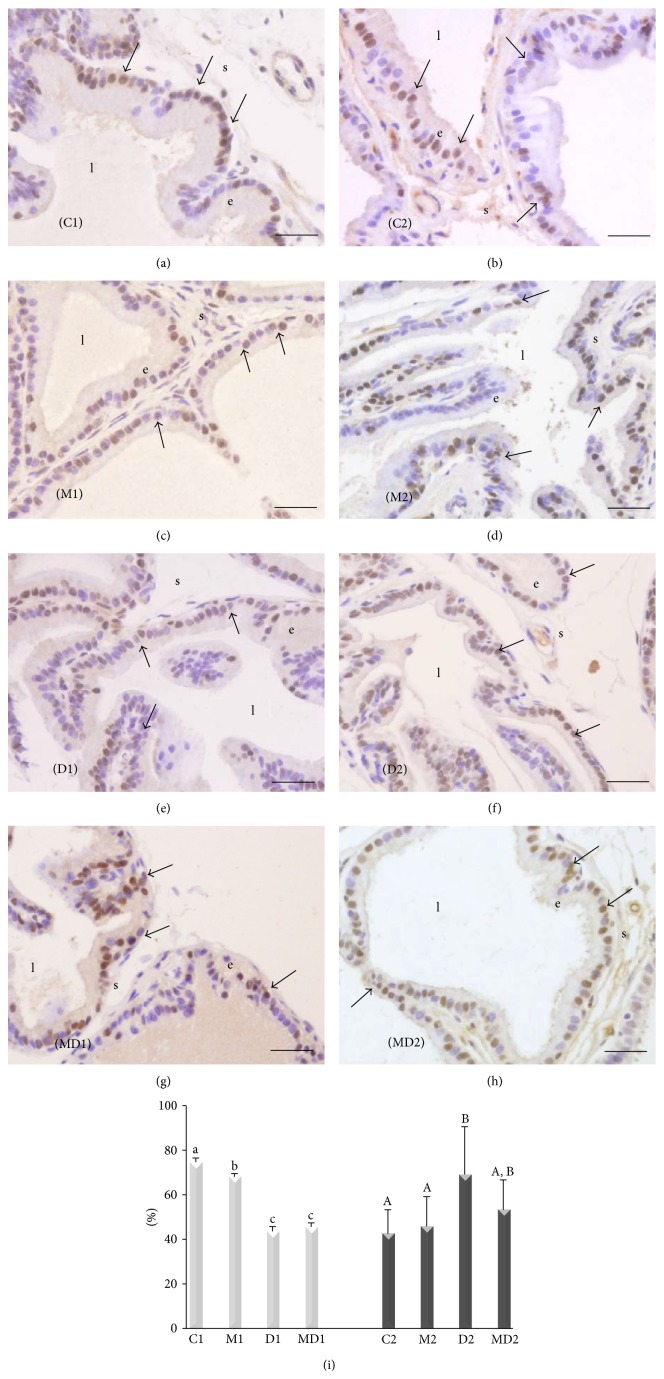
Immunohistochemistry ((a)–(h)) for the androgen receptor and quantification of AR-positive cells (i) in the acinar epithelium of rat ventral prostate (brown nuclei). (a) Short-term control (C1); (b) short-term control treated with MLT (M1); (c) short-term untreated diabetic (D1); (d) short-term diabetic treated with MLT (MD1); (e) long-term control (C2); (f) long-term control treated with MLT (M2); (g) long-term untreated diabetic (D2); (h) long-term diabetic treated with MLT (MD2). e: epithelium; l: lumen; s: stroma; arrow: AR-positive cells. Magnification: 400x, bar = 25 *μ*m. (i) AR-positive epithelial cells frequency (%); light bars: short-term experiment and dark bars: long-term experiment (*N* = 5 animals/group). Different lowercase letters indicate significant differences between experimental groups C1, M1, D1, and MD1 (parametric data), and different capital letters indicate significant differences between groups C2, M2, D2, and MD2 (nonparametric data).

The treatment of healthy rats with MLT from weaning to 9 weeks reduced the number of AR-positive cells in the prostatic epithelium at early adulthood (*p* = 0.03; Figures 2(c) and 2(i)). However, the frequency of AR-positive cells did not change when the treatment was extended for an additional 7 weeks (Figures 2(d) and 2(i)). One week of diabetes decreased the AR immunostained cells by 40% compared to group C1 (Figures 2(e), 2(g), and 2(i)), and MLT treatment did not prevent this depletion (*p* < 0.0001). Following two months of diabetes, the proportion of cells expressing AR nearly doubled (40%), and MLT consumption abrogated this increase (Figures 2(f), 2(h), and 2(i)).

#### 3.1.3. Proliferation, Apoptotic Rates, and PCNA Content

**Figure 3:**
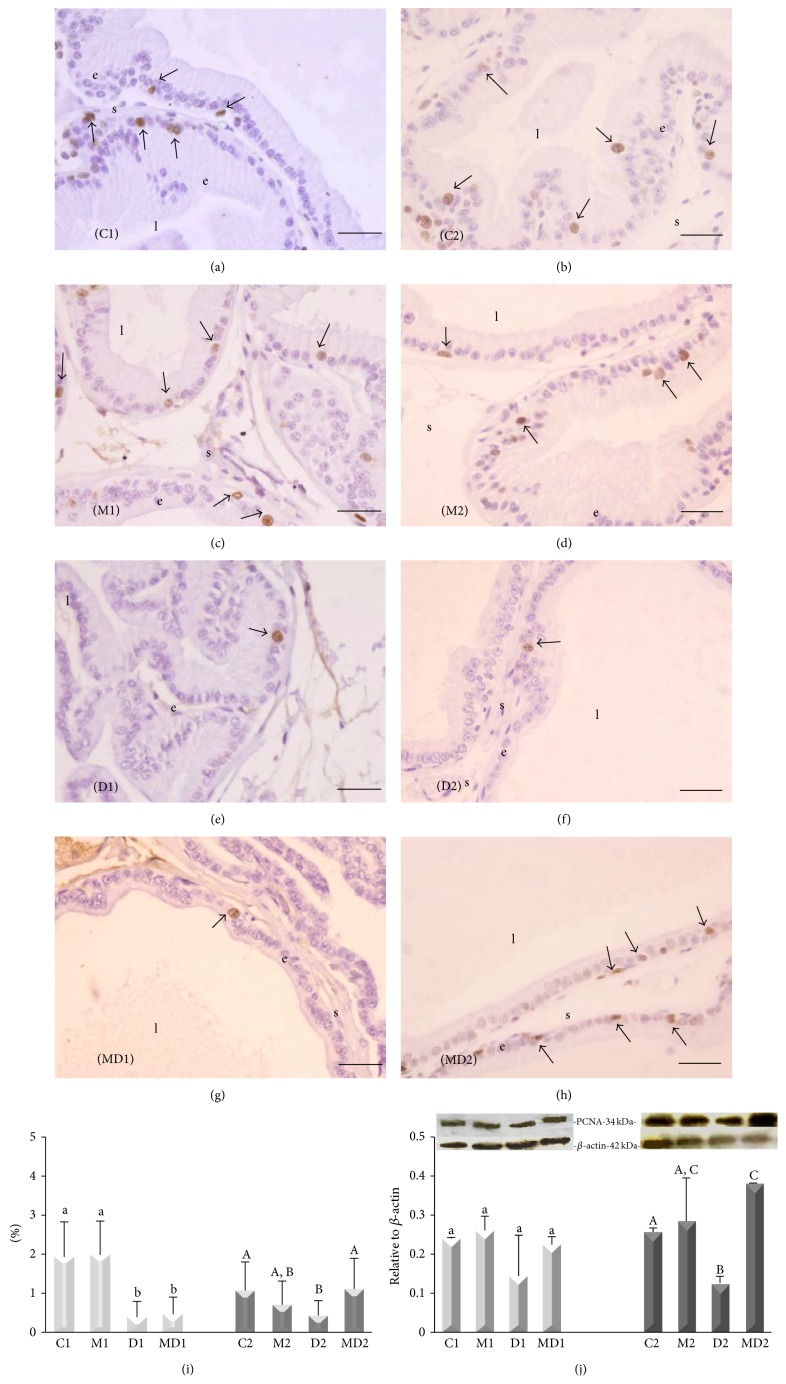
Proliferation index, evaluated by immunocytochemistry for proliferating cell nuclear antigen (PCNA) and Western blotting. (a) Short-term control (C1); (b) short-term control treated with MLT (M1); (c) short-term untreated diabetic (D1); (d) short-term diabetic treated with MLT (MD1); (e) long-term control (C2); (f) long-term control treated with MLT (M2); (g) long-term untreated diabetic (D2); (h) long-term diabetic treated with MLT (MD2). e: epithelium; l: lumen; s: stroma; arrow: PCNA-positive cells (brown nuclei). Magnification: 400x, bar = 25 *μ*m. (i) Relative frequency (%) of PCNA-positive cells. (j) Western blotting for PCNA in ventral prostate extracts and the respective densitometry analysis. *β*-actin was used as internal control loading. Light bars: short-term experiment and dark bars: long-term experiment (*N* = 5 animals/group). Different lowercase letters indicate significant differences between experimental groups C1, M1, D1, and MD1 (parametric data: PCNA protein content; nonparametric data: frequency of PCNA-positive cells), and different capital letters indicate significant differences between groups C2, M2, D2, and MD2 (parametric data: PCNA protein content; nonparametric data: frequency of PCNA-positive cells).

The frequency of cell proliferation did not alter in the prostates of groups M1 and M2 (Figures 3(a), 3(b), 3(c), 3(d), and 3(i)). Cell proliferation in the gland was reduced by 80% after one week and by 40% after two months of diabetes (Figures 3(e), 3(f), and 3(i)). MLT therapy prevented the decrease in cell proliferation in the prostate caused by long-term, but not short-term diabetes (Figures 3(g), 3(h), and 3(i)). Western blotting analysis showed no variation in PCNA expression in the short-term experiment; however, higher expression of PCNA was detected (60%) for group MD2 compared to group D2 in the long-term experiment (Figure 3(j)).

**Figure 4:**
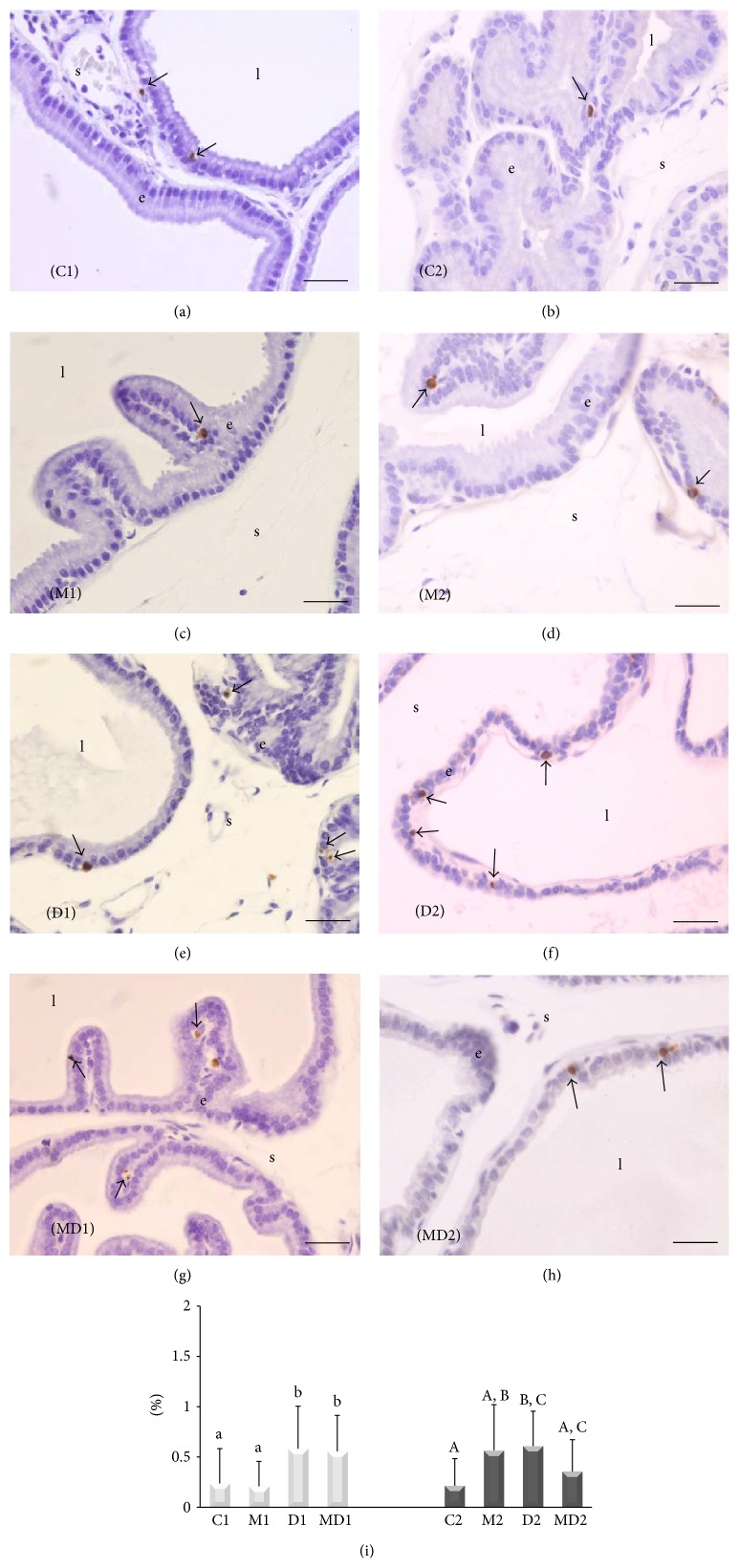
Apoptotic cells quantified by the TUNEL method (brown nuclei). (a) Short-term control (C1); (b) short-term control treated with MLT (M1); (c) short-term untreated diabetic (D1); (d) short-term diabetic treated with MLT (MD1); (e) long-term control (C2); (f) long-term control treated with MLT (M2); (g) long-term untreated diabetic (D2); (h) long-term diabetic treated with MLT (MD2). e: epithelium; l: lumen; s: stroma; arrow: PCNA-positive cells (brown nuclei). Magnification: 400x, bar = 25 *μ*m. (i) Relative frequency of apoptotic cells (%). Light bars; short-term experiment and dark bars: long-term experiment (*N* = 5 animals/group). Different lowercase letters indicate significant differences between experimental groups C1, M1, D1, and MD1 (nonparametric data), and different capital letters indicate significant differences between groups C2, M2, D2, and MD2 (nonparametric data), according to ANOVA followed by the Tukey (*post hoc*) or Kruskal-Wallis test followed by Dunn's test (*post hoc*).

The administration of MLT did not affect apoptosis in the prostate of healthy rats in both experiments, although there was a nonsignificant augmentation in group M2 (Figures 4(a), 4(b), 4(c), 4(d), and 4(i)). The number of apoptotic cells increased by two-fold in the gland of diabetic animals (Figures 4(e), 4(g), and 4(i)). MLT ameliorated the levels of apoptosis in the prostate of rats after two months of diabetes (Figures 4(h) and 4(i)).

#### 3.1.4. Immunolocalization and Quantification of MTR1B

**Figure 5:**
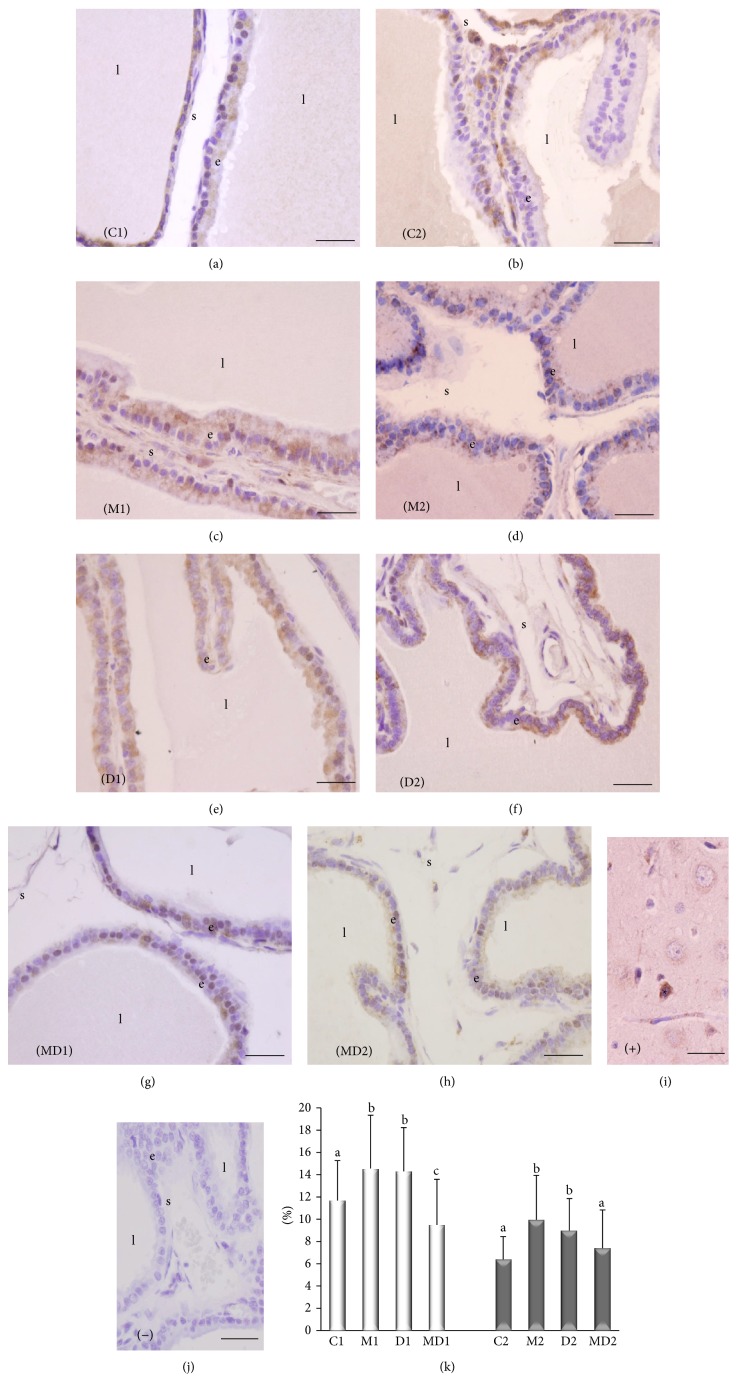
Tissue localization of melatonin receptor type 1B (MTR1B) in the ventral prostate of rats. (a) Short-term control (C1); (b) short-term control treated with MLT (M1); (c) short-term untreated diabetic (D1); (d) short-term diabetic treated with MLT (MD1); (e) long-term control (C2); (f) long-term control treated with MLT (M2); (g) long-term untreated diabetic (D2); (h) long-term diabetic treated with MLT (MD2). (i) Negative control; (j) positive control, brain tissue section of rat. (k) Relative frequency (%) for MTR1B-positive areas. e: epithelium; l: lumen; s: stroma. Magnification: 400x, bar = 25 *μ*m. Light bars: short-term experiment and dark bars: long-term experiment (*N* = 5 animals/group). Different lowercase letters indicate significant differences between experimental groups C1, M1, D1, and MD1 (nonparametric data), and different capital letters indicate significant differences between groups C2, M2, D2, and MD2 (parametric data), according to ANOVA followed by the Tukey (*post hoc*) or Kruskal-Wallis test followed by Dunn's test (*post hoc*).

The immunolocalization of melatonin receptor type 1B occurred specially in the prostate epithelium (Figure 5). Tissue sections of rat brain were used as a positive control and the neuron cell bodies presented positive staining for MTR1B (Figure 5(i)). The relative frequency of stained areas was increased by ~30% due to melatonin administration to healthy rats and to experimental diabetes (Figures 5(c)–5(f) and 5(k)). MLT avoided such augmentation of relative frequency of MTR1B stained areas in diabetic animals of both experiments (Figures 5(g), 5(h), and 5(k)).

### 3.2. *In Vitro* Results

#### 3.2.1. Effects of MLT and HG Medium on Cell Proliferation

**Figure 6:**
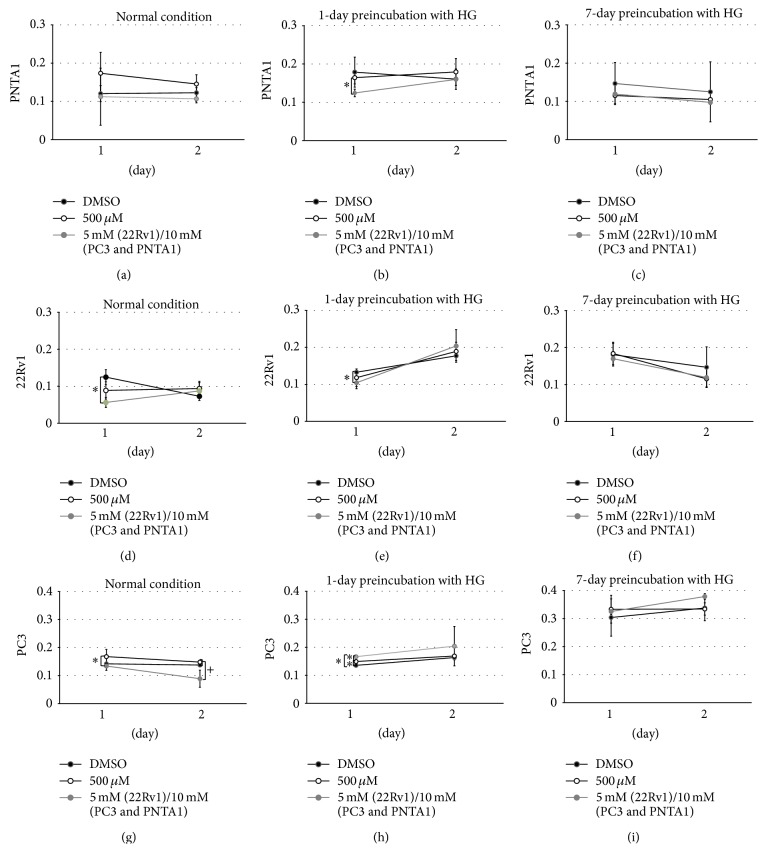
MTT assay (absorbance values) for cell proliferation for PNTA1 ((a), (b), and (c)), 22Rv1 ((d), (e), and (f)), and PC3 cells ((g), (h), and (i)) under normal conditions ((a), (d), and (g)), 1 day of preincubation with hyperglycemic medium ((b), (e), and (h)), 7 days of preincubation with hyperglycemic medium ((c), (f), and (i)), and treatment with DMSO, 500 *μ*M, 5 mM, or 10 mM of MLT for 1 and 2 days. *∗* represents significant differences among the different concentrations of melatonin exposure for 1 day (parametric data). + represents significant differences among the different concentrations of melatonin exposure for 2 days (parametric data).

PNTA1 cells were not affected by MLT under NC after 24 or 48 h of incubation (Figure 6(a)). The short incubation with HG medium and concomitant treatment with MLT at 10 mM decreased the amount of mitosis (*p* = 0.02; Figure 6(b)). This effect was not observed in the 7-day preincubation experiment (Figure 6(c)).

Cancer cell line 22Rv1 exhibited a decrease in cell proliferation (*p* = 0.008) after 1 day of exposure to 5 mM MLT under NC (Figure 6(d)). The same exposure period to MLT, but with a previous incubation of one day in HG medium, provoked the same effect, decreasing the cell proliferation by 23% with 5 mM (Figure 6(e)). These cells exhibited an early increase in proliferation after a short preincubation with the HG condition (Figure 6(e)) and an inverse pattern after a long preincubation, decreasing the viability to approximately 40%, independently with MLT treatment in both time intervals (Figure 6(f)).

Exposure to the lowest MLT concentration under NC for 24 h favored PC3 cell proliferation. However, after 48 h, MLT at the highest molarity diminished PC3 viable cells by 60% (Figure 6(g)). The preincubation of PC3 cells in HG medium for one day had a synergistic action associated with MLT on the proliferation index after 24 h in a dose-dependent manner (Figure 6(h)). This effect was enhanced 48 h after the addition of MLT, mainly at the 10 mM concentration. One week of previous exposure to HG augmented PC3 cell proliferation, compared to 1 day of previous incubation, regardless of the treatment with neurohormone, especially after 2 days of 10 mM MLT exposure (Figure 6(i)).

#### 3.2.2. Cell Cycle Analysis by Flow Cytometry

**Figure 7:**
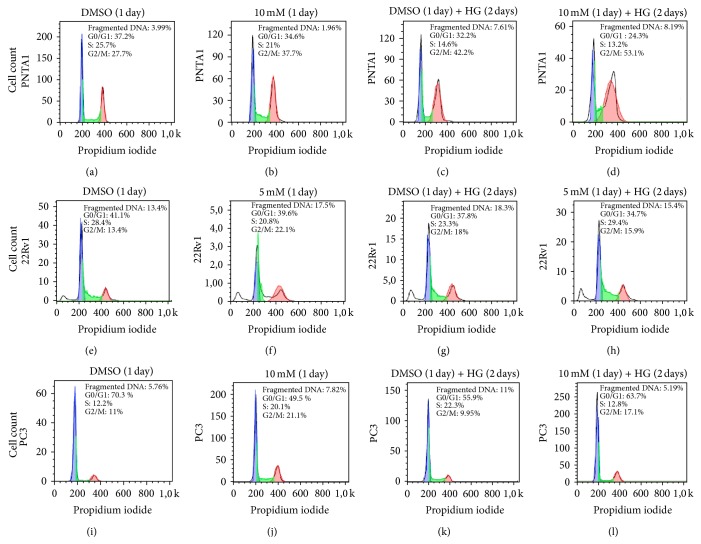
Determination of cell cycle analysis by DNA content using flow cytometry. 22Rv1 ((a), (b), (c), and (d)), PNTA1 ((e), (f), (g), and (h)), and PC3 ((i), (j), (k), and (l)) cells maintained under normal conditions ((a), (e), and (i)), treated with MLT (5 mM or 10 mM) for 1 day ((b), (f), and (j)), incubated with HG medium for 2 days ((c), (g), and (k)), and preincubated with HG medium for 1 day and then exposed to MLT (5 mM or 10 mM) for an additional 1 day ((d), (h), and (l)). DNA fragmentation: white peaks; G0/G1: blue peaks; S: green areas; and G2/M: red peaks.

The 1-day exposure of PNTA1 cells to 10 mM MLT under NC increased the population of G2/M cells by 36% in comparison to control medium (Figures 7(a) and 7(b)). The incubation of these cells with HG for 24 h increased the G2/M cell population by 52% (Figure 7(c)), and this increase was higher (92%) following 24 h of MLT incubation (Figure 7(d)). However, there was a 90% increase in the number of cells with DNA fragmentation when this cell line was incubated in HG medium and a 105% increase when incubated in HG medium and MLT (Figure 7(d)). There was a decrease in cell population at the S phase in both groups incubated with HG and a reduction in the G0/G1 cell population after HG and MLT incubation (Figure 7(d)).

Melatonin treatment of 22Rv1 cells cultivated in NC for 24 h increased the percentage of apoptosis by 30%, the number of cells in interphase by 28%, and the number of G2/M phase cells by 64% (Figures 7(e) and 7(f)). The exposure of 22Rv1 cells to HG medium also increased the apoptotic (36%) and G2/M (34%) cells (Figures 7(e) and 7(g)), and the addition of MLT attenuated these alterations (Figure 7(h)).

In the PC3 cell line, MLT caused a decrease of 30% in cells in the G0/G1 phase (Figures 7(i) and 7(j)) and an augmentation of 92% in G2/M cells. HG conditions did not affect the proliferation (G2/M) of this lineage but increased the number of apoptotic cells by 90% (Figure 7(k)). Such alterations were restored by MLT (Figure 7(l)).

## 4. Discussion

In addition to corroborate with previous evidence that diabetes markedly reduces cell proliferation and increases apoptosis in the prostate [37, 39, 43, 53], the present results indicate, for the first time, that MLT administration (10 *μ*g/kg body weight/day) to Wistar rats retrieved the proliferative activity of the prostate after two months of experimental diabetes. This retrieval was associated with improvement of the androgen synthesis that is known to be impaired in chronic diabetes [43, 45, 53]. In addition it was observed that prostate response to MLT treatment differed during the disease progression, following alterations of testosterone levels, because modifications of proliferation and apoptosis in the gland or improvement of circulating testosterone was not found in the animals after short-term diabetes. We also noted that MLT administration to healthy rats since weaning had a negative action on testosterone synthesis but did not interfere with proliferative and apoptotic response in prostate. This suppressive action of MLT on androgen levels of healthy rats was expected and it may be due to the modulation of gonadotropin-inhibitory hormone (GnIH) and its respective receptor [59], the decrease in LH levels [32], or the direct action of MLT in interstitial testicular cells through melatonin receptors [60]. Presumably, no effect was verified on cell proliferation and apoptosis rates because testosterone reduction did not induce a conspicuous change in AR expression in the gland of MLT-treated healthy rats, as indicated by a slight decrease of 10% in the frequency of AR-positive cells in short-term experiment. Then, our data indicated that, even at lower doses than usually employed previously [6, 61, 62], the proliferative and antiapoptotic effects of MLT treatment in the prostate of animals with chronic diabetes are likely due to its indirect action on circulating testosterone levels and also reflected the improvement of androgenic action in the gland under diabetes as demonstrated by AR-positive cells frequency. The increase of serum testosterone levels in diabetic animals treated with melatonin should be analyzed carefully, especially when melatonin is intended to be applied as a coadjutant in the treatment of prostate cancer for patients with metabolic disturbances, such as diabetes.

Furthermore, testosterone influence in the prostate response to MLT in diabetes was also triggered by conventional membrane receptor. MTR1 had been related to antiproliferative response of prostate cancer cells to MLT, which leads to the downregulation of activated AR signaling and upregulation of p27^kipi^ in 22Rv1 cells [63]. Our immunohistochemical data showed a significant reduction of melatonin receptor type 1B (MTR1B) for MD2 group in relation to D2 group, which can support the increased proliferation index found in these animals. In addition, in a parallel investigation with a similar experimental protocol, we demonstrated that MLT had effective antioxidant action in this gland under diabetes by equilibrating glutathione S-transferase (GST), catalase, and glutathione peroxidase (GPx) activities [64]. The MLT ability to attenuate the production of reactive species (ROS) and also regulating the expression of proteins of the apoptotic pathways, such as Bcl-2 and Bax [65, 66], have been related to the antiapoptotic property of this neurohormone. The increase in PCNA content in the melatonin-treated diabetic group (MD2), besides expressing an increase in cell proliferation, can also corroborate the protective action of MLT because PCNA is also involved in DNA damage repair and epigenomic maintenance [67]. Taken together the* in vivo* results demonstrate that response of diabetic prostate to the MLT treatment was mainly due to the alterations in androgen levels, as expected, but they also suggest the involvement of MTR1B receptor. Most evidence in the literature points to an antiproliferative action of MLT on prostate cancer cells, and* in vivo* studies with normal cells are scarce [14, 68, 69]. Therefore our results for the long-term diabetic group treated with MLT are in contrast to* in vitro* studies and they also indicate a differential action of MLT during normoglycemia or hyperglycemia.

To better clarify the implications of MLT from its secondary effect on androgen levels, and the interferences of hyperglycemia and AR expression, we examined MLT influence on cell proliferation of human prostate epithelium cells and prostate cancer cell lineages. It is known that MLT can detain mitosis through the attenuation of calcium influx induced by dihydrotestosterone (DHT) and downregulation of androgen signaling due to the nuclear exclusion of AR [17, 21, 22]. The inhibition of tumor enlargement due to the antiproliferative action of MLT is mediated by cell cycle delay at the G0/G1 phase and a shorter duration in the S phase by reducing the levels of cyclin D1 [16, 18, 19]. The usage of both tumor and nontumor cell lines was pertinent because it was observed that 8% of diabetic animals presented prostate carcinoma and 15% presented high grade intraepithelial neoplasia (data not shown). The glucose concentration used in the medium (450 mg/dL) is equal to the glycemic index of diabetic animals in the present investigation. Our study was performed with high MLT concentrations (5 mM and 10 mM) compared with most previous studies, which utilized doses in the nM and *μ*M range [21, 24, 25, 63]. Higher MLT concentrations were expected to be effective in concomitantly covering three different cell lines. The uptake of MLT by prostate cancer cells is poor and dependent on the cell cycle phase, and the cells in the present investigation were not synchronized. Therefore, high concentrations of MLT in the medium do not necessarily correlate with higher internal concentrations [29]. Thus, the solubility of MLT was hampered, and a higher concentration of DMSO had to be used to dissolve it.

Proliferation assays did not indicate a drastic antiproliferative and proapoptotic action of MLT under NC, as reported previously [70]. These attenuated unintended effects may be due to the shorter incubation times employed here. Sainz et al. [71] disposed the indoleamine for 2–6 days, allowing more time for its metabolism. Hevia et al. [72] proved that melatonin is still available in the medium and not transformed into its metabolites by prostate cancer cells until 24 h of incubation. Moreover, the intracellular levels of melatonin decay after 24 and 48 h [29], which support the possibility of differences in the influence of MLT between short and continuous exposure.

PNTA1 are human nontumoral cells that were used here as an analogous for the epithelium cells of rat ventral prostate. As observed* in vivo*, MLT did not elicit an antiproliferative response in PNTA1 cells under normal conditions and HG medium increased the percentage of fragmented DNA. As also observed in a previous research, there was no increase in proliferation by the association of PNTA1 cells and DMEM with HG (450 mg/dL) [73]. Androgen receptor positive but not androgen sensitive cells, 22Rv1, were highly sensitive to MLT in NC, particularly at high concentrations, but when incubated in HG medium, the MLT was capable of reducing cell proliferation of 22Rv1 after a short incubation period. PC3 cells revealed a different behavior from the other cells exhibiting a late antiproliferative response to MLT in NC and a synergistic proliferative effect with high glucose medium, as showed by both MTT and flow cytometry data. Thus, the prolonged incubation with HG medium impaired the mitosis of 22Rv1 and PNTA1 cells but improved this parameter for PC3 cells. Hevia et al. [74], using LNCaP and PC3 cells incubated under HG concentrations, observed an antiproliferative effect of MLT that was caused via modulation of glucose transporter type 1 (GLUT1). Our results do not completely agree with the above findings, particularly in the case of PC3 cells. These discrepancies can be explained by the different incubation times and concentrations of HG.

Although the use of* in vivo* and* in vitro* experiments allowed observing some parallelisms in the results, the differences between the microenvironments in which these cells are inserted should not be neglected. In this way, the* in vivo* experiment has a much more complex panel of variables. The prostate secretory epithelium is composed of various cell types, interrelated with each other, as the basal cells, transit-amplifying cells, intermediate cells, and the luminal cells [75]. Thus, none of the cell lines used in this research can represent the population heterogeneity of prostatic epithelium. It is worthy to mention that short-term diabetes drastically reduced the frequency of AR-positive cells in the prostate and this effect probably persists at later stages of the disease as previously reported by our research group after one-month of streptozotocin-induced diabetes [53]. Therefore the results observed for the longer duration experiment reflect the MLT influence in cells whose majority does not express AR. In this context, PC3 cells displayed a comparable behavior to the prostatic cells under chronic diabetes. Furthermore, in this case, the MLT response* in vitro* can be mediated by other signaling pathways, such as those related to specific membrane or nuclear receptors for this hormone.

## 5. Conclusion

Our* in vivo* data indicated that under chronic diabetes exogenous melatonin had a proliferative and antiapoptotic effect due its indirect action on testosterone circulating levels and also direct effect through specific MTR1B receptor. PC3 cell line showed a similar result to the* in vivo* experiments, because an increase in mitosis was elicited at longer preincubation times with hyperglycemic medium and MLT. As PC3 cells are androgen-independent prostate cancer cells, our findings indicate that this improvement of PC3 cell viability probably is not related to AR expression.
